# Perspectives and experiences of new migrants on health screening in Sweden

**DOI:** 10.1186/s12913-015-1218-0

**Published:** 2016-01-15

**Authors:** Faustine Kyungu Nkulu Kalengayi, Anna-Karin Hurtig, Annika Nordstrand, Clas Ahlm, Beth Maina Ahlberg

**Affiliations:** 1 Department of Public Health and Clinical Medicine, Division of Epidemiology and Global Health, Umeå University, SE- 901 87 Umeå, Sweden; 2 Norrbotten County Council, Public Health Center, SE-971 89 Luleå, Sweden; 3 Department of Clinical Microbiology, Division of Infectious Diseases, Umeå University, SE- 901 87 Umeå, Sweden; 4 Department of Women’s and Child Health, Uppsala University, SE- 751 85 Uppsala, Sweden; 5 Skaraborg Institute of Research and Development, SE-541 30 Skövde, Sweden

**Keywords:** Migrants, Screening, Infectious diseases, Interpretive description, Language barriers, Thematic analysis, Qualitative interview, Health policy, Migration policy, Sweden

## Abstract

**Background:**

In Sweden, migrants from countries considered to have a high burden of certain infectious diseases are offered health screening to prevent the spread of these diseases, but also identify their health needs. However, very little is known about their experiences and perceptions about the screening process. This study aimed at exploring these perceptions and experiences in order to inform policy and clinical practice.

**Method:**

Using an interpretive description framework, 26 new migrants were interviewed between April and June 2013 in four Swedish counties. Thematic analysis was used to analyze data.

**Results:**

The three themes developed include: new country, new practices; new requirements in the new country; and unmet needs and expectations. Participants described what it meant for them to come to a new country with a foreign language, new ways of communicating with caregivers/authorities and being offered health screening without clarification. Participants perceived health screening as a requirement from the authorities to be fulfilled by all newcomers but conceded that it benefits equally the host society and themselves. However, they also expressed concern over the involvement of the Migration Board staff and feared possible collaboration with health service to their detriment. They further stated that the screening program fell short of their expectations as it mainly focused on identifying infectious diseases and overlooked their actual health needs. Finally, they expressed frustration over delay in screening, poor living conditions in reception centers and the restrictive entitlement to care.

**Conclusions:**

Migrants are aware of their vulnerability and the need to undergo health screening though they view it as an official requirement. Thus, those who underwent the screening were more concerned about residency rather than the actual benefits of screening. The issues highlighted in this study may limit access to and uptake of the screening service, and compromise its effectiveness. To maximize the uptake: (1) linguistically and culturally adapted information is needed, (2) other screening approaches should be tried, (3) trained medical interpreters should be used, (4) a holistic and human right approach should be applied, (5) the involvement of migration staff should be reconsidered to avoid confusion and worries. Finally, to improve the effectiveness, (6) all migrants from targeted countries should be offered screening and efforts should be taken to improve the health literacy of migrants and the living conditions in reception centers.

## Background

### Migration to Sweden

Trends in migration to Sweden reflect the overall increase in migration internationally [1]. Sweden, traditionally a country of emigration turned into a country of immigration between the 1930s and the 1940s. Since then, immigration to Sweden has increased following the international trend and its patterns have changed over time depending on immigration policy and various events around the world [2, 3]. Nowadays, the migration of people in need of protection is on the increase due to the growing number of countries in conflict and social inequalities in the world. However, the role of globalization should not be underestimated as it facilitates social and economic interconnections between nations through advances in communications technology, the decline in travel costs, transnational trading zones which encourage the freedom of movement of people and the emergence of a global labor market. Globalization also plays an important role in reinforcing disparities between rich and poor countries fueling the movement of workers and people in search of better lives from the former to the latter [4]. According to the Swedish Migration Board, an increasing number of European Union (EU) citizens could easily move to Sweden from 2001 for temporary or long-term jobs as Sweden became part of the Schengen cooperation area [2, 3]. Moreover, since 2009, labor migration from non-EU countries started to increase again after a drastic reduction in the 1970s, as a result of a new law that opened the Swedish labor market for immigrants from outside the EU and European Economic Area (EEA) states [3]. According to Statistics Sweden, the country is now a diverse society where foreign born persons constitute over 16 % of the population [2, 3]. However, despite its substantial contribution to labor force and population growth, migration generates complex challenges for those who move and those who receive them [3–5].

### Migrants’ situation in Sweden

Depending on how they enter Sweden, migrants have different statuses that give them different rights and access to public services, including health services [6]. In this regard, asylum seekers and irregular migrants are the most vulnerable groups of migrants in Sweden. Besides living in limbo, they have limited rights compared to quota refugees and other immigrant groups who have a residence permit with regard to housing, access to care and other public services [7]. For instance, apart from children under 18 years who are entitled to full care, care for asylum seekers is regulated by the Act (2008:344) on Health Care for Asylum Seekers and ‘others’ (*Lagen om Hälso- och sjukvård åt asylsökande m.fl.*) that limits their entitlements to “care that cannot wait”, maternity care, care in relation to abortion and family planning [8]. Their daily allowance varies between 50 and 71 SEK /day per person (7 - 10 US dollars) and they can either arrange their own accommodation, for example, with relatives or friends, the so-called EBO (*eget boende*) or get a temporary accommodation at one of the Migration Board accommodation facilities (ABO: *anläggningsboende*) while they await a decision on their application [7, 9]. In the latter case, they are housed at one of the facilities by the Migration Board staff where, families often share a flat while several single persons share a room [7, 9]. Irregular migrants are extremely disadvantaged as they are ineligible for most public funds and services and live in constant fear of deportation. Until recently, they had no rights to mainstream health services and had to pay out of pocket if they use it or rely on care provided by non-governmental organizations and informal networks of health professionals [8]. But, since July 2013, they have the same entitlement to care as asylum seekers, but in practice they face many difficulties [10].

At the same time, evidence suggests that migrants from countries with high prevalence of certain infectious diseases such as Human Immunodeficiency Virus (HIV), tuberculosis (TB) and hepatitis B are disproportionately affected by these diseases even in receiving countries in the EU and EEA [11]. Parallel trends in reported cases and the number of new migrants from these countries have been considered to pose particular challenges for health care systems and have raised concern over the spread of these diseases [5, 11–14]. Meanwhile, migrants, particularly new migrants may face multiple barriers in accessing care, which may increase their vulnerability to these infections [5, 14, 15]. Consequently, 16 of the 28 EEA countries including Sweden have adopted legislations and implement interventions such as the health screening programs that target newly arrived migrants from countries considered to have high burden of infectious diseases in order to contain the diseases and mitigate potential impact on public health, individual migrants as well as long-term social and health consequences [12, 13, 16, 17].

### The Swedish health screening program for new migrants

The Swedish National Board of Health and Welfare (NBHW) regulations urge each county council to offer adult and child ‘*migrants’*, health screening soon after arrival in Sweden in accordance with the Act (2008:344) on Health Care for Asylum Seekers and ‘others’ (*Lagen om Hälso- och sjukvård åt asylsökande m.fl.*) and the Act (2013:407) on Health Care for certain aliens residing in Sweden without necessary authorization (*Lagen om hälso- och sjukvård till vissa utlänningar som vistas i Sverige utan nödvändiga tillstånd*) [18]. According to the regulations, the screening serves two different goals, namely to identify health problems requiring immediate attention and prevent the spread of infectious diseases of public health significance through early (within two months) diagnoses, treatment, prevention and care in accordance with the Communicable Disease Act (2004:168) [18]. The latter requires physicians to treat and report all diagnosed cases to the County medical officer. Likewise, all potentially sick persons are under obligation to seek medical attention for diagnosis, treatment and to assist clinicians with contact tracing for prevention. All health care and medication is free for the patient [19]. Additionally, the screening is expected to provide information to new migrants regarding the Swedish health care system and their entitlement to medical and dental care [12, 18]. However, as mentioned before, asylum seekers and irregular migrants are only entitled to care that cannot be postponed, suggesting that not all health problems are taken care of.

The NBHW guidelines further emphasize that health screening shall include (a) information on the purpose of the health screening, (b) the contact details of the caregiver who will do it, (c) that the screening is voluntary and (d) that an interpreter will be used when needed. Additionally, the guidelines emphasize the importance of providing information about the offer in a language that the recipient understands and remind at least once if the offer was declined or not received [18]. In practice, information about health screening offer is given by the asylum case officer (asylum seekers) or the integration coordinator (quota refugees) at the first visit to one of the Swedish Migration Board and integration offices respectively. Thereafter, the Migration Board staff/integration coordinator inform and send the contact details of newcomers to the local migrant/refugee coordinator (*flyktingsamordnare*) at the respective county council, who in turn, further conveys the contact details to the respective units in primary care [7]. An appointed practitioner nurse at the respective care unit then send written invitations for screening by post to the newcomers and book an interpreter for the visit as all persons who are not fluent in Swedish are legally entitled to an interpreter when dealing with the authorities [20]. According to the NBHW, the screening shall include: an interview about the newcomer past and current physical and mental health, vaccination status and exposure to risk of infection, and other information that may be needed from infection control standpoint. Additionally, a physical examination and blood tests may be performed if necessary [18]. Subsequently, the county council may apply for compensation for performed health screenings at the Migration Board if the screening is carried out within 12 months from the date the migrant first settled in the county [12]. However, the NBHW guidelines are often complemented by local guidelines and recommendations from the county councils. In addition, it is not clear whether the concepts of *‘migrants’* and ‘others’ (*m.fl*.) mentioned in the NBHW guidelines also include students, those coming through family ties and migrant workers [18]. Thus, it is up to each county to offer screening to other migrant categories or not.

Although widespread in host countries, screening new migrants has been criticized as being ineffective, discriminative, stigmatizing and of poor value [21, 22]. One of the criticisms is that there is no clear evidence about the individual and public health benefits or cost-effectiveness of screening migrants from countries considered to be endemic for certain infectious diseases [22]. Additionally, despite consensus on the utility of screening, there are differences in the implementation and practices among countries. These variations include target groups, timing and locations of screening [13, 22]. Furthermore, the screening often takes place only once at the time of initial entry and whether this could prevent onward transmission if the individual is infected or in the case of visits to the home country is unclear. It is also argued that screening is not beneficial to certain migrants, given that they are not followed up or provided with effective, timely and uninterrupted curative and preventive care [22].

While compulsory screening is a common policy in some countries, health screening is voluntary in Sweden although it can be compulsory under the Communicable Disease Act [13, 18, 19]. Despite that annual statistics from the Swedish Association of Local Authorities and Regions (*Sveriges Kommuner och Landsting*) show low uptake rate nationally with variations between counties with counties in the north performing better than most counties in southern Sweden [23]. In 2013, only 43 % of the 54 259 registered asylum seekers in Sweden underwent health screening. This has raised concern over the implementation of the program and its effectiveness [24]. However, while the screening has been implemented for decades in Sweden, the views of migrants remain unknown. Studies focusing on migrants’ views about the screening are quite rare in Sweden. Thus, this study aimed at exploring the perceptions and experiences of new migrants targeted for health screening, particularly their understanding, concerns and ideas about the screening service in order to inform policy and clinical practice.

## Methods

### Research design

We adopted an interpretive description (ID) approach to investigate migrants’ experiences of and perspectives on health screening to generate knowledge that could inform practice, decision making and policy development. According to Thorne and colleagues, ID aims to answer questions of relevance to a clinical discipline that inevitably occur in the real world of health care practice. Thus, the desired outcome of ID is knowledge that changes practice rather than theory development or general qualitative description [25]. The research questions that guided this study include a range of issues around the health screening process: perceptions of health screening, sources of information, reasons for undergoing the screening, expectations from screening and perceptions of using interpreters.

### Study setting and participants

Participants were purposively recruited by the first author (FKNK) at selected primary health care centers, Swedish for Immigrants (SFI) Schools and reception facilities (accommodation centers) for asylum seekers in four counties in Northern Sweden. The selection criteria were, being an immigrant from one of the countries considered to have high burden of certain infectious diseases, and living in Sweden for no more than five years. Nurses who commonly screen migrants facilitated contact with potential participants who were then approached and asked if they would like to participate after informing them about the study. The recruitment continued until no new ideas were generated. The 26 participants were predominantly asylum seekers (*N* = 20) and the majority were still waiting for the decision about their asylum application. The remaining six were refugees (3) and family ties (3). The participants came from 14 different countries outside the European Union. Table 1 displays participant characteristics.

**Table 1:** Socio-demographic characteristics of study participants

Geographic origin	Sex: Men/Women	Age ranges (Years)	Education (Years)
Africa			
Eritrea	3 /3	27–51	6–12
Democratic Republic of Congo	1/1	28–40	6–8
Kenya	0/1	29-	10
Sudan	2/0	27–52	3–15
Somalia	0/3	21–29	0–5
Asia			
Afghanistan	2/1	28–34	4–5
Irak	0/1	42-	10
Syria	1/0	29-	12
Mongolia	0/1	25-	10
Kyrgystan	1/0	41-	10
Palestina	1/0	38-	8
Pakistan	0/1	29-	12
Europe			
Russia	1/ 1	27–36	10
Albania	1/0	38	8
All participants	13/13	21–52	0–15

### Data collection

The first author interviewed the 26 participants who agreed to participate in the study between April and June 2013 with each interview lasting for 45–60 min. Most interviews were carried out face-to-face using telephone interpreters, except for ten participants who were fluent in or whose first language was among the five languages (English, French, Lingala, Swahili or Swedish) that the first author speaks fluently. The following open ended questions were used with follow up questions when unexpected issues arose: Could you please describe your first contact with the Swedish health care service? Why did you go to the health care service? What does health screening mean to you? How did you know about health screening? What happened during health screening? How does it feel to be assisted by an interpreter? Demographic data were also collected and included: age, sex, educational level, country of origin and type of migration. A Dictaphone was used to record each interview, which was thereafter transcribed verbatim by an independent transcriber and read through by the first author to inform the ongoing data collection process. Supplementary field notes and preliminary analytical notes were written and were appended to each interview transcript.

### Ethical issues

Apart from ethical approval from the Regional Ethical Committee at Umeå University, information about the study was translated both orally and in written form in each participant’s language with the help of international master and postgraduate students in Public Health at Umeå University who originated from participants’ home countries. Each interview began with an explanation of the purpose of the study. Oral consent was obtained prior to each interview and the voluntary nature of participation, the right to terminate participation at any moment without consequences or explanation was explained. All information collected was treated confidentially and the results are reported anonymously.

### Data analysis

**Fig. 1:**
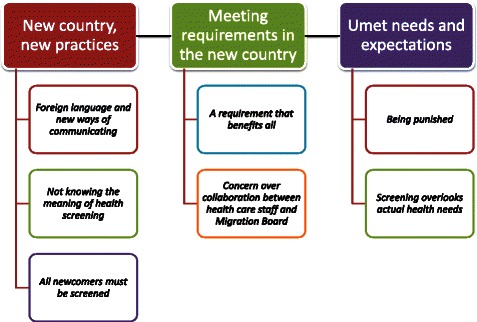
Main themes and subthemes. An illustration of the main themes and subthemes identified during the analysis

This inquiry was conducted by a bicultural (natives and migrants) and multi-professional research team (clinician, sociologist and public health professionals and interpreter). Thus, as with all qualitative research, data collection and analysis informed one another iteratively in this study. After the interview transcriptions were finalized, a thematic analysis approach was applied to the data/transcripts. The first and last authors read the transcripts and field notes separately, to familiarize themselves with data and manually coded its interesting features. These initial codes were thereafter compared and discussed to reach agreement on differences in meaning of codes and emerging patterns [26]. The other members of the research team (AKH, AN, CA) also participated in the discussions and acted as point of reference for the issue under exploration. Data relevant to each code were then collated and the meanings of codes were refined through the process. The different codes were then sorted into potential themes that gathered all relevant data for each potential theme, which were then reviewed through the ongoing analysis process to generate clear definitions of themes and sub-themes [26]. Finally, after refinement of themes, three themes with associated sub-themes that made sense of what migrants said about screening were developed: 1) new country, new challenges; 2) meeting requirements in the new country and 3) unmet needs and expectations. Figure 1 shows the main themes and subthemes.

## Results

Participants described what it meant for them to come to a new country with a foreign language, new ways of communicating with caregivers/authorities and being offered health screening without clarification. Participants perceived health screening as a requirement from the authorities to be fulfilled by all newcomers but expressed concern over the involvement of the Migration Board staff and the possible collaboration with health care providers to the detriment of migrants. Nevertheless, they acknowledged that health screening equally benefits the host society and themselves, and those who were not yet invited were disturbed. Yet, they also stated that the screening service fell short of their expectations as it mainly focused on identifying infectious diseases and overlooked their actual needs. They further expressed frustration over the delay in screening, poor living conditions in reception centers and a restrictive entitlement to care. These issues are highlighted in the following section with illustrative quotes from participants.

### New country, new practices

#### Foreign language and new ways of communicating

The participants spoke of the difficulties in moving to a new country and adapting to a new environment, a foreign language as well as a new way of communicating and interacting with the health care and other public officials. They said it was strange to receive information from the authorities including information about health screening offer by post in letters written in Swedish, a language they could not read or understand as expressed by one participant: “*In Sweden, when they call us at the hospital, the Migration Board or elsewhere, they write a letter. The fact that they send letters in Swedish is a big problem for many people” (IP2).*

Some participants mentioned using Google tool for online translation in an attempt to understand the content of the letter. Others said they consulted their countrymen or other migrants who had been screened before and could explain to them what to do. After receiving the letter they should go to the health care center and meet the “Migration Board’s” nurse. According to participants, most people missed their appointments because the letter was in Swedish rather than in their native languages, as stated by one participant:

*All the letters are in Swedish. This is a big problem for everyone. Most people go around with the paper and ask others: “Can you, please translate this for me”. We told the migration staff that this was a big problem for us because every time we have to go to our neighbors for translation and most of them don´t know Swedish very well either. So they cannot translate properly. They said: “No, it’s the rule of the immigration office to send letters only in Swedish and not in English or Dari”… (IP1).*

The participants moreover stressed that written information would still be an issue for those with poor literacy skills even when translated in appropriate languages. Even when given a guided tour by the migration staff on the first day, the participants still reported different opinion regarding finding their way to the health center. Those housed near health centers said they had no problem finding their way. However, for those living far, it was difficult to find the way to the health center afterwards, and due to language and literacy barriers, it was difficult to ask passers-by for help, as indicates below:

*They brought me with a car and showed me this place (the health center), but I was really tired and confused that day. It was difficult to find my way later on. But, fortunately my neighbor showed me the way to the clinic…. (IP1).*

*So when you cannot speak any of the local languages, it’s very difficult to ask people to show you where something is. So this is a problem, especially for those who are not enough educated and cannot understand the circumstances of where they are living (IP2).*

Another challenge mentioned was the difficulty to effectively communicate with health care professionals through an interpreter. This was considered to be complicated and a potential cause of misunderstanding. Some participants felt they got wrong interpreters even though they spoke the same language (did not pay attention to their dialects while booking interpreters), which led to misunderstandings as indicated below:

*Interpreters are always a problem. Sometimes they book interpreters from Iran for people from Afghanistan. I mean, there are some diseases and ailments we call in our special way in my country. I think there are some ways to call them in our language that Iranians do not know (IP8).*

Participants who were interviewed with the assistance of interpreters were more positive about them in contrast to those interviewed without interpreters. The latter described the interpreters as unskilled and unprofessional. While talking about interpreters, one participant who was fluent in English said, “*I do not like interpreters. They are playing with people’s lives. It often goes wrong because of interpreters. Most of them are disasters. They get angry when you say you do not understand” (IP3).* Some interpreters were even accused of breaching confidentiality. One participant who expressed concern over privacy said, “*I prefer a phone interpreter. It’s better when you talk about your health problems” (IP12)”.*

#### Not knowing the meaning of health screening

The participants indicated they did not know or understand why they had to go to the health center because even during the screening consultation, the nurses did not explain why they called them and what they were doing. According to the participants, the nurses simply draw blood without telling why the blood was taken. One participant said, “*They usually do not say what type of infection. They only say that we want to see if you have infection” (IP7).* Another participant who shared this view commented:

*I do not really know what they did. It is very important to know. If they call me today for the health screening, I will ask them why? They should tell us why they call us and explain what they do” (IP22).*

Those who reported asking the nurses why they took blood said they were told it would be used to test for tuberculosis, HIV and hepatitis, but the participants expressed frustration over not receiving the test results afterwards. Those who requested for test results were either told: “everything is fine” or they received a paper containing technical words written in Swedish. One participant shared, *“She did not tell me anything, but later on she gave me a paper with test results. Unfortunately, I did not understand the contents because of the language and medical terms (IP24)*.

#### All newcomers must be screened

The participants expressed the view that the health screening was a requirement from the authorities that all newcomers must meet and could therefore not decline the offer. One participant desperately said, “*I am the one who came to their country. We are not invited here so we should not complain about this (IP3).* Another participant who shared this view stated, “*I heard that everyone has to do it. Otherwise, you will get into trouble with the Migration Board” (IP1).* It was further explained that the Swedish authorities believed they carried dangerous diseases and should therefore be screened to contain the spread of these diseases and protect the population as the following quotes suggest:

*It is the system; everybody who comes to Sweden has to do it. You cannot say no, that is the rule. Every country has the right to be safe. Maybe some people have TB or HIV. The country has the right to do it (IP20).*

*The nurse told me that every newcomer must take these tests because people come from different countries and different environments. So they must be checked in Sweden…. She said ….all newcomers must do it…. (IP10).*

### Meeting requirements in the new country

#### A requirement that benefits all

Despite the perception that health screening was a constraint, all participants spoke positively about it and indicated this benefited not only the Swedish society, but also migrants. They were grateful to be given the opportunity to check their health status, which they could not do in their home countries due to different barriers. One participant said, “*It is the rule. But, at the same time it is good for my health. I get screened for free (IP21).* Another commented, *“I wanted to do it anyway because it’s important for me to know about my health status. I think they do it to protect me and the society (IP25).* Another participant who experienced traumatic event on his way to Sweden stressed he could not say no because he actually needed medical attention, “*Actually*, *it was good that they called me because I was beaten and tortured in Syria. I would just say yes (IP17).*

#### Concern over collaboration between health care staff and Migration Board

Most asylum seekers were concerned that the test results would affect their asylum application and consequently believed that the Migration Board and health care service worked closely together with respect to health screening. They felt the authorities wanted to know their health status including what serious diseases they may have before deciding whether they could stay or not. One participant angrily said, “*They tell the Migration Board everything about people’s health. How else would they know the people’s health statuses?”* (IP13). They further explained how the screening interview reminded them of the asylum interview at the Migration Board adding that the two services asked similar questions in order to cross-check their answers. One participant questioned, “*During the screening interview, they asked more than expected, they asked strange questions. The nurse asked me “why did you come to Sweden?” What does that have to do with my health? (IP12)*.

They moreover asserted that the screening nurses commonly known as *“asylsjuksköterska”* (asylum seekers’ nurse) and *flyktingsjusköterska* (refugees’ nurse) were not employed by the county council, but rather by the Migration Board. One participant reasoned, *“She said: ‘I do not work for the Swedish Migration Board’. But, they ask the same questions*” (IP12). Another participant shared the same view, “*Migration Board nurse. That is how we refer to her”* (IP3*)*. One participant that a nurse suspected never attended the health screening appointment despite reminders defended herself and said, “*I do not like to go there because 99 % of the people who work there are employed by the Swedish Migration Board” (IP11).* But, later during the interview, it became evident that she underwent health screening at a transit reception center in another setting, which the nurse was apparently not informed about. Another participant reported she was requested to undergo health screening for the second time after moving to a new area even though her medical records could prove she had done it before. Despite acknowledging the benefits of health screening, most asylum seekers found it strange to be asked by the Migration Board staff during the interview if they had a serious illness or if they wanted to or had undergone health screening. As a result, they became suspicious of the offer and argued that the health care staff should not call them. Rather they should be allowed to seek care by themselves when the need arose.

### Unmet expectations and needs

#### Being punished

The participants, particularly asylum seekers also talked about their unmet needs and expectations. Those who were housed in asylum reception centers complained about their living conditions, (which they equated with punishment). They described experiencing the overcrowding and poor sanitary conditions at the reception centers as a punishment and believed they were intentionally put in such conditions to force them back home and to deter other migrants coming to Sweden. One participant noted, “*Six people from different countries and with different cultures in one single room, it’s like punishment” (IP13).* Other participants living at a reception center in another setting gave similar accounts by pointing to the long distance between their accommodation centers and the health screening units as a reason why they were often late or missed their medical appointments. They felt frustrated over being required to pay for missed appointments and said this was unfair because they only received little money as a daily allowance as indicated below:

*We share a room and they cook for us. If you miss meals you may not have food for the day. That’s not what I expected. We live in the forest and it’s hard to be punctual for appointments at the hospital. There is no transportation in the area and we have to pay 250 Swedish crowns when we miss appointments. It takes three hours to get to the hospital. I recently missed an appointment to see the doctor because of that (IP26).*

The participants also expressed concern over the health risk posed by their living conditions, which was compounded by the long waiting time for screening appointment. Talking about delay in screening, one participant reasoned:

*One gets the letter to attend the health screening appointment like weeks after being in the refugee camps. This means that if you could infect others, they would probably become infected. I mean, it would be too late to stop it … (IP8).*

In the same vein, another participant who obtained Swedish residence permit in his home country on the basis of family ties expressed concern over not being called for health screening after a year in Sweden. Even though, he had not received any so far, he kept on waiting. He said he could not contact the health care service because his schoolmates at SFI said he would receive a letter. He added he could not understand that the same Swedish authorities who granted him residence permit in his home country were not aware (of his presence) he was already living in Sweden.

#### Screening overlooks actual health needs

The participants stressed that the health screening program was inadequate because it only focused on identifying infectious diseases and not their perceived health needs as indicated by this participant, “*They just look for infectious diseases and nothing else. I expected that they would take people’s health seriously. What most asylum seekers need help with is mental illness” (IP6).* Another participant added: “*It was good, but it was not enough. I expected more than that. Health screening means that they are going to check your entire body and not just look for infectious diseases” (IP2).*

The participants reported having received no health information though they felt in need of it. They also expressed a wish to repeat the health screening and argued that having the screening only once was not enough. One participant commented, “*If you are healthy today, it does not necessarily mean that you will be healthy throughout life. (IP14).* Another participant added, *“I want to repeat the tests every six months. Many things happen over the life course, you know (IP25).*

Asylum seekers were also disappointed for not receiving treatment after being diagnosed with certain health conditions. They were shocked to be told they had to wait until they got a residence permit before receiving appropriate treatment. One participant narrated in desperation:

*I was told that I have hepatitis, but I have not received treatment… I would like to get treatment for hepatitis. I do not want the disease to get worse. If she said that there was no treatment I would not worry…It is also important to get treatment and not just know that you have a disease (IP14).*

Consequently, they felt that the medical staff did not take their complaints seriously for which they argued they were not treated with respect. One participant said, “*If you have a residence permit, they treat you in a completely different way” (IP13).* Corroborating this view, another participant said*,” Residence permits makes a difference” (IP11).* They said that many people avoided seeking care even though they were in need of it because of staff attitude. One asylum seeker said that when he told the nurse that he did not understand what she was saying the nurse angrily replied in English and said, “*You’re in Sweden, and you should speak Swedish”(IP12).* Others were disappointed when they met a nurse as they expected to see a doctor or when they could not get prescriptions.

## Discussion

This study suggests that migrants, particularly asylum seekers acknowledge the need for health screening. However, because of the structural organization of the screening, they see health screening not only as a benefit but rather as a requirement they have to fulfill in order to get permission to stay in Sweden. The health screening process reinforces the current debates and the “othering” of migrants across the EU where they are constantly represented as a threat to the native population. Thus, the screening process normalizes the idea that immigration is a source of danger and racialize and construct immigrants from countries considered to pose health risks as ‘diseased others’ and a threat to the nation. In this way, health screening can be understood as a context where various markers of difference including, race, legal status, citizenship, intersect to restrict admission to the country, social rights and access to care. In other words, health screening can be interpreted as a proxy for discrimination based on national origin, which is a common aspect of racial discrimination.

This study highlights a number of issues that impact on the screening process. These include lack of (cultural and linguistic) sensitivity and inclusiveness of the screening service, health care staff attitudes, a focus on infectious diseases that overlook migrants’ actual needs and the involvement of staff from the Migration Board that contributes to the perception that the screening is a legal requirement. Finally, the poor living conditions, delay in screening combined with restrictive entitlement to care for asylum seekers is counterproductive as it may increase theirs and other migrants’ vulnerability to infectious diseases and poor health, and further expose them to discrimination. If not addressed these issues may limit the uptake of the screening service, prevent early detection and effective management of ill health and thereby undermine the achievement of public health goals.

This study indicates that the screening service is not easily accessible to migrants because of structural barriers mentioned above as well as the way information about the screening offer is conveyed for example, the use of Swedish language which has been previously identified as a structural discrimination by health care staff who participated in previous studies [15, 27]. This practice, in addition disregards the NBHW guidelines (that stipulate that information about health screening should be sent in a language migrants understand), and violates the administrative Act (*Förvaltningslagen para. 8*) that recommends that all public services use interpreters when dealing with people with limited competence in Swedish [18, 20]. Moreover, new migrants might not understand the rationale for screening as they may have symptom- driven health seeking behaviors [15, 27]. Participants in this study stressed that some migrants missed their appointment because they could not read or understand the content of the letter and thus did not know what to do, where to go for screening after receiving the letter or simply had reservations about providers inviting them for screening. Lack of awareness as well as inability to access available information on health matters and services may prevent effective utilization and cause delay in diagnosis and treatment of ill health as illustrated by the late diagnosis of HIV, cancers and diabetic complications commonly reported among migrants [11, 28, 29]. Lack of cultural sensitivity, language differences, limited literacy and unfamiliarity with the host countries’ healthcare system are well known barriers that limit migrants’ access to and use of health care, including the uptake of screening service in many host countries [15, 28–31].

Language and cultural differences have also been reported as serious problems in the interaction of migrants with health care staff [15, 27, 29, 30]. Even in this study, communication problems were reported during the medical interview despite the use of interpreters. This was said to be caused by the interpreters’ lack of competence, professional misconduct such as breach of confidentiality as well as the screening staff inattention to language or dialect variation while booking interpreters. Poor interpretation can compromise symptom reporting and lead to misunderstandings with increased risk for wrong diagnoses, inappropriate treatment and a frustrating encounter for both clinician and patient as reported in other studies [15, 27, 32]. However, it is worth stressing that only participants who were not assisted by interpreters during data collection talked negatively about them suggesting that those who were dependent on interpreters were afraid of negative consequences and refrained from criticizing them. Poor communication between migrants and health care staff might as well reflect lack of cultural competence among providers, low health literacy among migrants as well as differences in cultures and expectations about screening combined with lack of information about the Swedish health care system and health-related rights, which possibly generate a feeling of discrimination and disrespect leading to dissatisfaction and mistrust towards medical staff [15, 27, 28, 30]. Equally important, is the use of technical terms and “medical jargon” by the screening staff that confused migrants and could negatively affect the care [33].

Nevertheless, participants complained that the screening staff did not provide enough information about the screening process, screened diseases or the results of blood tests, which made them anxious as indicated in findings from a previous study that insufficient explanation by screening staff raised anxieties about the process [21]. This stands in contrast with the NBHW guidelines on provision of information to new migrants [18] and raises the question of whether informed consent was obtained. In a previous study with migrant students, no association was found between undergoing screening and level of knowledge among migrants or their attitudes towards TB, which was actually low with negative attitudes. This led to the conclusion that health professionals missed the screening opportunity to improve TB knowledge and to change attitudes among this vulnerable population [34]. These findings suggest the need to assess migrants’ knowledge and understanding of screening and screened diseases in order to address misconceptions, emphasize the benefits of screening, decrease anxiety, improve acceptability and the image of health services and thereby facilitate future utilization [27].

Participants also mentioned a number of issues that may be unique to asylum seekers, including fear of the consequence of a positive test, confidentiality issues, poor living conditions and limited access to care, which generated frustration and feeling of discrimination and disrespect. Even though participants saw health screening as a benefit for their health, they regarded themselves as passive recipients as they believed it was an official requirement they had to fulfill without questioning. They perceived undergoing screening as a way to abide by the rules, and thus feared that a positive result could compromise their asylum application [27]. As a result, those who underwent the screening were more concerned about residency rather than the actual benefits of health screening. Fear of legal consequences of a positive result has been previously described as barrier to migrants’ use of screening and HIV testing services in host countries [27, 31, 35]. This issue was exacerbated by the involvement of the staff from the Migration Board in the screening process raising concerns about the possibility of information sharing. Fear of links between health providers and immigration authorities has been suggested as an important factor that may deter migrants from seeking care, compromise the development of a trustful patient- provider relationship and limit access to and use of available services [27]. Contrary to widely held beliefs, a previous survey of legal migrant students showed that this issue is not limited to asylum seekers. Although all respondents had permission to stay in Sweden, fear of deportation was the most important determinant of reluctance to seek HIV/AIDS care [35]. Conversely, fear of legal consequences was found in a recent study to be a subsidiary barrier for use of HIV testing service by Latino migrants in Spain. The authors attributed this contrasting finding to the equal access that both natives and migrants (regardless of legal status) had until recently, and warned that this situation might be reversed by the new Spanish austerity policies regarding healthcare for migrants [36]. In other words, guaranteeing equal access to care may promote the use of available services by migrants.

This study further stresses the role of housing and dispersal policy for asylum seekers in impeding their access to available services including the screening service and exacerbating their vulnerability to poor health. Participants who were housed at the Migration Board reception centers complained about their precarious living conditions, particularly their housing situation that they experienced as a punishment. Those who were housed in remote areas, far from the nearest health care unit, reported facing difficulties in accessing the screening service and health care in general due to lack of transport, its costs or unfamiliarity with their new environment, leading to delayed or missed appointments. These issues need further exploration to find the best way to offer the screening service. Moreover, participants worried that their overcrowded conditions coupled with the long waiting time for screening and test results might increase their risk of acquiring and spreading infectious diseases and potentially jeopardizing the effectiveness of the screening [27]. There is evidence to suggest that the poor housing conditions actually increase the risk of transmission of infectious diseases among migrants as indicated in a report about an outbreak of TB at an asylum seekers’ reception center in Sweden [37]. Similar issues were noted in Greece where a report from the Médecins Sans Frontières (MSF) suggested that more than 60 % of health problems among asylum seekers and migrants were directly caused by or linked to the inhumane living and hygienic conditions in detention facilities [38]. However, fear of infection reported in this study might as well express the stigma related to screened diseases prevailing within migrant communities, which may also partly explain the mistrust towards interpreters [21, 28, 31, 34, 35].

Participants also reported that the screening service fell short of their expectations as it only focuses on identifying infectious diseases of public health significance while overlooking their actual health needs. In this way, the screening policy lacked a holistic perspective on health and was thus perceived as a discriminatory device against their ethnicity or citizenship. Similar findings were reported in our previous study with health care staff who described the issue as a dilemma and potential source of conflicts [27]. Besides, the focus on infectious diseases is inconsistent with the NBHW guidelines that emphasize not only the detection of infectious diseases of public health significance, but also the need to identify physical and mental health problems requiring medical attention in new migrants [18]. Lack of perceived medical benefits may negatively affect acceptance and utilization of the screening service [28]. Meanwhile, previous reports have clearly pointed out that contrary to migrant workers, asylum seekers and refugees are more vulnerable to mental illnesses, most screening programs tend to focus only on infectious diseases and fail to address the disparities in health and care needs prevailing between and within migrant groups [39, 40]. This contributes to the perception that migrants are infectious disease threats to the host population. These laws and practices to control and prevent the spread of communicable diseases may reinforce fear of contagion and create a hostile environment by portraying migrants as disease vectors, and lead to their discrimination within the health care system and the wider society [27]. Some participants reported unfriendly experiences during the medical encounters or when they attempted to seek care corroborating findings from other studies [28, 31]. A recent study conducted in the UK suggested that discrimination from health professionals was an important barrier to the use of screening service by migrants [31]. Moreover, screening only migrants may also create a false sense of security among native residents that can hamper prevention efforts [41].

Furthermore, contradictory policies, such as offering health screening to asylum seekers while restricting their entitlements to care can be counterproductive to public health as it may delay care, increase the severity of diseases and its subsequent costs [17]. Asylum seekers who participated in this study were confused and frustrated by the paradox of being offered health screening, but denied access to treatment for conditions that were not perceived as immediate threats to life or public health because of their legal status. This can intensify worries and suffering among people who are already in a vulnerable situation. In addition, this restrictive policy is in conflict with human rights law and the Health and Medical Care Act (1982:763), which emphasizes equal access for all [8, 42] and represents a form of institutional discrimination that reflects an attempt to make migration unattractive. Such conflation of public health with migration issues raises ethical issues, creates a dilemma for health professionals [27, 42] and constitutes a serious impediment to access timely care leading to advanced disease and significant social and health consequences [17]. Similar legislations in Australia did not only result in poor health outcomes, but also deter refugees from undergoing health screening, which increased concerns about the spread of communicable diseases among them, and this in turn, exacerbated their stigmatization [43].

Finally, like in other EU countries, the screening policy mainly targets asylum seekers and does not encompass all categories of migrants. Not including all migrants from targeted countries may compromise the effectiveness of the screening program as evidence suggests that migrants are more likely to be infected with HIV or TB after migration through contacts with fellow countrymen and women [37, 44]. For instance, we could not identify any migrant worker (a growing group) or student among participants who had been screened in this study. Even though family ties and quota refugees can also be offered health screening under certain circumstances, only the screening of asylum seekers and undocumented migrants is regulated by law [10, 45]. Thus, the Swedish Migration Board is required to provide information (contact details) only about asylum seekers and is under no obligation to inform the county councils about other categories such as family ties, quota refugees, students or labor migrants regardless of the National Strategy to combat HIV/AIDS and other Communicable Diseases of public health significance [46]. This ambiguity and conflict in laws also makes it difficult for health care staff to reach all new migrants from targeted countries and may partly explain why the family ties participants who were waiting for the invitation letter did not get it [27]. This can also be (mis)interpreted as having a residence permits removes a migrant from the need to be screened. Furthermore, as mentioned earlier, the NBHW guidelines are unclear as to whether the screening should be offered to all migrants from targeted countries or some specific groups of migrants [18]. Thus, some counties choose to offer the screening to all migrants from targeted countries, whereas others limit the offer to some subgroups depending on whether the cost of screening will be covered by the State or not [27]. For instance, migrant workers and students are not routinely offered the screening in most counties, whereas undocumented migrants who are not officially registered may be hard to reach or deterred from responding to the screening offer for fear of being reported to the migration authorities. Moreover, although asylum seekers are one of the most vulnerable subgroups of migrants, singling them out as the only category to be screened may expose them to social stigma and discrimination associated with their legal status, their countries of origin and screened diseases [21, 28, 31]. This may prevent them using the screening service. From a public health perspective, there is a need for a new way of thinking and approaching migration and health issues. Contrary to the threat of disease approach that has been traditionally used, more attention should be paid to the complex and multiple factors that increase the vulnerability of migrants to poor health. Changing paradigm will not only benefits migrants, but also the society as a whole. Future research is, thus, necessary to better understand the role of screening in improving the health of migrants, and ultimately the health of all.

### Limitations of the study

There are a number of limitations that deserve mention. Although studies involving human participants are context bound and, findings from this study may only apply to the Swedish or similar contexts, our findings are congruent with findings from other studies and provide basis for improving the screening program [21, 27, 31, 47]. Moreover, preliminary results were presented and discussed with migrants who participated in a workshop on the health screening of new migrants organized by the National Federation of Immigrant Women’s Associations (*Riksförbundet Internationella Föreningar för Invandrarkvinnor - RIFFI)*, which supported our interpretation of the data. Although we used a purposive sample, we sought a maximum diversity to reflect the wide range of countries targeted with screening. However, undocumented migrants might have other specific barriers and were therefore not included in this study. Even if we were unable to identify a large number of migrants who declined screening, the issues raised in this study are likely to affect their decisions. Further work is needed to identify the determinants of screening uptake. The use of interpreters may have affected the results, but apart from criticism of interpreters’ competence, we found similar views with or without interpreters across the four settings and in feedback from migrant women during the workshop. Finally, we had discussions throughout the study and during the analysis process, which enhanced the interview guide, style as well as data interpretation.

## Conclusions

Migrants are aware of their vulnerability to poor health and the benefits of screening though they might perceive it as a requirement. However, structural and organizational issues highlighted in this study may negatively affect access to, acceptability and uptake of the screening service, and compromise its effectiveness. To maximize the uptake: (1) linguistically and culturally adapted information is needed to make the screening service accessible and acceptable to migrants, (2) other approaches such as outreach screening services at community centers, asylum reception centers, Swedish language schools or churches should be tried, (3) trained medical interpreters should be used during the screening interview, (4) a holistic and human right approach should be applied to the screening program in combination with other initiatives to address the health and care needs of new migrants, (5) the involvement of migration staff in the screening process should be reconsidered to avoid confusion and worries that may decrease acceptability and willingness to use the screening service. Finally, to improve the effectiveness, (6) all migrants from targeted countries should be offered screening and efforts should be made to improve the living conditions in reception centers as well as the health literacy of migrants through the screening process.

## Abbreviations

ABO: Anläggningsboende (*reception centre*)
EBO: Eget Boende (*Own or self-arranged accommodation*)
EEA: European Economic Area
EU: European Union
HIV: Human Immunodeficiency Virus
ID: Interpretive Description
MSF: Médecins Sans Frontières (*Doctors without borders*)
NBHW: National Board for Health and Welfare
RIFFI: Riksförbundet Internationella Föreningar för Invandrarkvinnor *(the National Federation of Immigrant Women’s Associations)*
SFI: Svenska för Invandrare (*Swedish for Immigrants*)
TB: Tuberculosis
